# Cytokine-mediated activation of human ex vivo-expanded Vγ9Vδ2 T cells

**DOI:** 10.18632/oncotarget.17498

**Published:** 2017-04-28

**Authors:** Eisuke Domae, Yuya Hirai, Takashi Ikeo, Seiji Goda, Yoji Shimizu

**Affiliations:** ^1^ Department of Biochemistry, Osaka Dental University, Hirakata, Osaka 5731121, Japan; ^2^ Department of Biology, Osaka Dental University, Hirakata, Osaka 5731121, Japan; ^3^ Department of Oral Science, Graduate School of Dentistry, Kanagawa Dental University, Yokosuka, Kanagawa 2388580, Japan; ^4^ Department of Laboratory Medicine and Pathology, Center for Immunology, Masonic Cancer Center, University of Minnesota Medical School, Minneapolis, Minnesota 55455, USA

**Keywords:** γδ T cells, IL-12/IL-18, IκBζ, STAT4, NF-κB p65

## Abstract

Vγ9Vδ2 T cells, the major subset of the human peripheral blood γδ T-cell, respond to microbial infection and stressed cells through the recognition of phosphoantigens. In contrast to the growing knowledge of antigen-mediated activation mechanisms, the antigen-independent and cytokine-mediated activation mechanisms of Vγ9Vδ2 T cells are poorly understood. Here, we show that interleukin (IL) -12 and IL-18 synergize to activate human ex vivo-expanded Vγ9Vδ2 T cells. Vγ9Vδ2 T cells treated with IL-12 and IL-18 enhanced effector functions, including the expression of IFN-γ and granzyme B, and cytotoxicity. These enhanced effector responses following IL-12 and IL-18 treatment were associated with homotypic aggregation, enhanced expression of ICAM-1 and decreased expression of the B- and T-lymphocyte attenuator (BTLA), a co-inhibitory receptor. IL-12 and IL-18 also induced the antigen-independent proliferation of Vγ9Vδ2 T cells. Increased expression of IκBζ, IL-12Rβ2 and IL-18Rα following IL-12 and IL-18 stimulation resulted in sustained activation of STAT4 and NF-κB. The enhanced production of IFN-γ and cytotoxic activity are critical for cancer immunotherapy using Vγ9Vδ2 T cells. Thus, the combined treatment of ex vivo-expanded Vγ9Vδ2 T cells with IL-12 and IL-18 may serve as a new strategy for the therapeutic activation of these cells.

## INTRODUCTION

Human Vγ9Vδ2 T cells predominate in the population of peripheral blood γδ T cells and play a key role in immunity against microbial infection and tumors [1]. The Vγ9Vδ2 T cell receptor (TCR) recognizes a conformational change in butyrophilin 3A1 induced by the association with phosphoantigens such as isopentenyl pyrophosphate (IPP) [2–5]. IPP is produced as an intermediate of the mevalonate pathway, and the dysregulation of this pathway in transformed cells or the pharmacological inhibition by aminobisphosphonate causes an accumulation of IPP in tumor cells, which leads to their killing by activated Vγ9Vδ2 T cells [6].

Vγ9Vδ2 T cells express natural-killer group 2, member D (NKG2D) and recognize stress-induced self ligands such as MHC class I-related chains A and B (MICA/B) and UL-16 binding proteins (ULBPs) [7]. Co-stimulation by these ligands through NKG2D enhances Vγ9Vδ2 T cell-mediated cytotoxicity against tumor cells [7]. In concert with TCR and NK-receptor stimulation, inflammatory cytokines such as IL-12 and IL-18 provide a critical signal to promote effector functions such as cytotoxicity and IFN-γ expression and enhance the clonal expansion of Vγ9Vδ2 T cells [8–11].

In conventional αβ T cells and NK cells, antigen-independent, cytokine-mediated activation serves as an alternative regulatory mechanism. In CD8^+^ T cells, antigen-independent activation mediated by combined exposure to IL-12 and IL-18 is an important aspect of their sentinel response to a local microbial infection before the development of the adaptive immune response [12]. The most intensely studied effect of IL-12 and IL-18 on CD8^+^ T cells is the profound induction of IFN-γ, and the combined treatment of IL-12 and IL-18 induces proliferation of virus-specific memory CD8^+^ T cells [13]. NK cells are also a target of IL-12 and IL-18 [14], and the presence of these cytokines might be critical for controlling MHC class I-deficient tumors [15]. Thus, the antigen-independent, cytokine-mediated activation of lymphocytes is an important mechanism that regulates adaptive and innate immunity.

In contrast to the growing knowledge regarding antigen-mediated activation mechanisms of Vγ9Vδ2 T cells, knowledge regarding antigen-independent and cytokine-mediated activation mechanisms is considerably less. In the present study, we examine the alternate activation mechanisms of human ex vivo-expanded Vγ9Vδ2 T cells using inflammatory and homeostatic cytokines. We demonstrate that Vγ9Vδ2 T cells produce IFN-γ and upregulate activation markers in response to combined exposure to IL-12 and IL-18. Moreover, combined exposure to IL-12 and IL-18 induced the proliferation of Vγ9Vδ2 T cells independent of antigen, which was enhanced upon subsequent exposure to IL-2 or IL-15. Further, cytokine-activated Vγ9Vδ2 T cells produced higher levels of granzyme B and efficiently killed tumor cells. Finally, our results address the induction of IκBζ and the sustained activation of STAT4 and NF-κB as pivotal mechanisms for the IL-12/IL-18-induced activation of Vγ9Vδ2 T cells.

## RESULTS

### Vγ9Vδ2 T cells produce IFN-γ in response to combined exposure to IL-12 and IL-18

**Figure 1:**
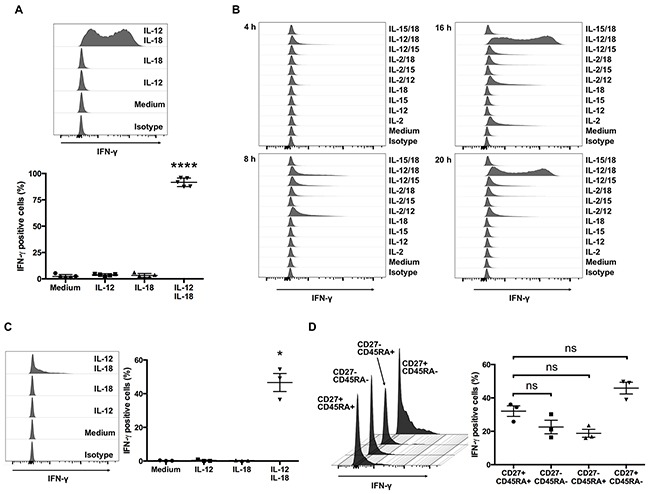
Human Vγ9Vδ2 T cells produce IFN-γ in response to IL-12 and IL-18 **(A)** Ex vivo-expanded human Vγ9Vδ2 T cells were cultured for 16 h with medium alone, IL-12 (10 ng/mL), IL-18 (10 ng/mL), or IL-12 and IL-18 (10 ng/mL each). Cells were harvested and analyzed to detect the cell-surface of CD3 and Vδ2 TCR and intracellular expression of IFN-γ. Data from healthy donors (n = 5) obtained from independent experiments. Bars represent the mean and standard error of the mean (SEM), ****P < 0.0001, one-way ANOVA, followed by Tukey's multiple comparison test. **(B)** Ex vivo-expanded Vγ9Vδ2 T cells were stimulated with indicated cytokines for 4, 8, 16 and 20 h and the cells were analyzed in the same way as **(A)**. **(C)** Freshly isolated human Vγ9Vδ2 T cells were stimulated and analyzed in the same way as **(A)**. **(D)** IL-12/IL-18 treated fleshly isolated Vγ9Vδ2 T cells were further analyzed for the differentiation status based on the cell surface expression of CD27 and CD45RA. Data from healthy donors (n = 3) obtained from independent experiments. Bars represent the mean and SEM, *P < 0.05, one-way ANOVA, followed by Tukey's multiple comparison test.

The synergistic effect of IL-12 and IL-18 on IFN-γ production by NK and αβ T cells is well established and may have a pathophysiological role [13, 14]. Although ex vivo-expanded Vγ9Vδ2 T cells produce IFN-γ in response to phosphoantigen [16], to the best of our knowledge, there are no studies that show whether Vγ9Vδ2 T cells produce IFN-γ in response to combined exposure to IL-12 and IL-18. To address this question, we treated Vγ9Vδ2 T cells with IL-12, IL-18, or both for 16 h and then tested for intracellular IFN-γ. Although IL-12 or IL-18 alone did not induce detectable production of IFN-γ, combined treatment with IL-12 and IL-18 considerably induced IFN-γ production in ex vivo-expanded Vγ9Vδ2 T cells (Figure 1A).

Next, to determine whether the ability of cytokines to induce robust expression of IFN-γ in Vγ9Vδ2 T cells is unique for the combination of IL-12 and IL-18, we treated ex vivo-expanded Vγ9Vδ2 T cells with combinations of cytokines including IL-2, IL-12, IL-15, and IL-18. Although treatment with IL-2 alone, IL-2/IL-12, or IL-12/IL-15 induced IFN-γ production, the duration and intensity was limited compared with IL-12/IL-18-induced IFN-γ production (Figure 1B). Thus, the combination of IL-12 and IL-18 is a highly potent activator of ex vivo-expanded Vγ9Vδ2 T cells.

Because the Vγ9Vδ2 T cells used in these experiments were pre-activated in the process of expansion by IL-2 and zoledronic acid, we next examined whether freshly isolated human Vγ9Vδ2 T cells also produce IFN-γ in response to IL-12 and IL-18. Although the percentage of IFN-γ positive cells was not high as seen in ex vivo-expanded Vγ9Vδ2 T cells, combined treatment of IL-12 and IL-18 also induced IFN-γ production in freshly isolated human Vγ9Vδ2 T cells (Figure 1C). Freshly isolated Vγ9Vδ2 T cells are a heterogeneous population of naïve cells and antigen experienced cells (Supplementary Figure 1). To determine if previous antigen exposure affects IFN-γ production in Vγ9Vδ2 T cells, we analyzed cell surface expression of CD27 and CD45RA in IL-12/IL-18 treated freshly isolated Vγ9Vδ2 T cells. We did not observe significant differences between naïve (CD27^+^ CD45RA^+^) phenotype population and antigen experienced populations (Figure 1D). These results show that combined exposure to IL-12 and IL-18 induces IFN-γ expression in Vγ9Vδ2 T cells, and the pre-activation of Vγ9Vδ2 T cells during ex vivo expansion results in enhanced IFN-γ production in response to these cytokines.

### IL-12 and IL-18 synergize to induce the proliferation of ex vivo-expanded Vγ9Vδ2 T cells

**Figure 2:**
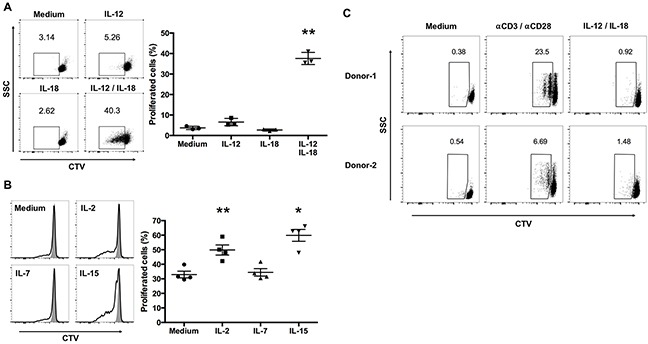
IL-12 and IL-18 synergize to induce the proliferation of ex vivo- expanded human Vγ9Vδ2 T cells **(A)** CellTrace Violet (CTV)-labeled ex vivo-expanded Vγ9Vδ2 T cells were stimulated as described in Figure 1, washed extensively, and cultured in complete medium without cytokines for 3 days. Dot plots were gated on CD3^+^Vδ2^+^ cells and CTV^low^ cells were calculated as proliferating cells (left). The data were obtained from 3 independent experiments each using Vγ9Vδ2 T cell from different donors (right). **(B)** CTV-labeled ex vivo-expanded Vγ9Vδ2 T cells were stimulated with IL-12/IL-18 for 16 h, washed extensively, and cultured for 3 days with medium alone, IL-2 (10 U/mL), IL-7 (1 ng/mL) or IL-15 (1 ng/mL). Histograms were gated on CD3^+^Vδ2^+^ cells and CTV^low^ cells were calculated as proliferating cells (left). The data were obtained from 4 independent experiments each using Vγ9Vδ2 T cells from different donors (right). Bars represent the mean and SEM, *P < 0.05, **P < 0.01, one-way ANOVA, followed by Tukey's multiple comparison test. **(C)** CTV-labeled freshly isolated human Vγ9Vδ2 T cells were stimulated with IL-12/IL-18 or anti-CD3/anti-CD28 antibodies for 3 days. Dot plots were gated on CD3^+^Vδ2^+^ cells and CTV^low^ cells were calculated as proliferating cells.

Upon antigen stimulation, lymphocytes start producing effector molecules, change their pattern of expression of cell-surface activation markers, and start proliferating [17]. Further, IL-12 and IL-18 treatment induces the proliferation of murine virus-specific CD8^+^ memory T cells without antigen stimulation [13]. We hypothesized that IL-12/IL-18 treatment might also induce Vγ9Vδ2 T cells to proliferate. Therefore, we assessed cytokine-mediated Vγ9Vδ2 T cell proliferation using the CellTrace Violet (CTV) dilution assay. Similar to virus-specific CD8^+^ memory T cells, treatment with IL-12/IL-18 induced the proliferation of ex vivo-expanded Vγ9Vδ2 T cells in the absence of antigen stimulation (Figure 2A). Treatment with IL-12 or IL-18 alone did not induce the proliferation of Vγ9Vδ2 T cells (Figure 2A).

Cytokines that engage common cytokine receptor γ-chains play crucial roles in the survival and proliferation of lymphocytes [18]. We therefore determined the ability of IL-12/IL-18-activated Vγ9Vδ2 T cells to proliferate in the presence of low concentrations of IL-2 (10 U/mL), IL-7 (1 ng/mL), or IL-15 (1 ng/mL). IL-2 and IL-15, but not IL-7, enhanced the proliferation of IL-12/IL-18-activated Vγ9Vδ2 T cells (Figure 2B).

Next, we examined the effect of IL-12 and IL-18 on the proliferation of freshly isolated Vγ9Vδ2 T cells. Although anti-CD3 and anti-CD28 treated positive control cells induced CTV dilution, IL-12/IL-18 treatment did not induce CTV dilution in freshly isolated Vγ9Vδ2 T cells. Thus, in contrast to the induction of IFN-γ, the proliferation of freshly isolated Vγ9Vδ2 T cells is not induced by the combined treatment of IL-12 and IL-18 (Figure 2C).

These results suggest that IL-12 and IL-18 program ex vivo-expanded Vγ9Vδ2 T cells to proliferate and that this effect is further enhanced by subsequent exposure to IL-2 or IL-15.

### Ex vivo-expanded Vγ9Vδ2 T cells treated with IL-12 and IL-18 express increased levels of effector molecules and decreased levels of co-inhibitory receptors

**Figure 3:**
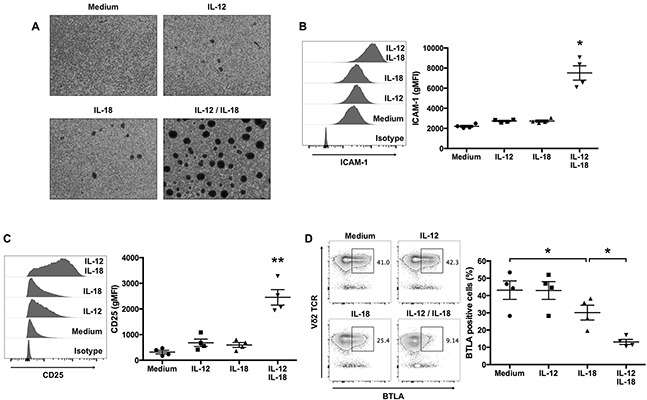
IL-12 and IL-18 treatment of human Vγ9Vδ2 T cells enhances ICAM-1 and CD25 expression, and inhibits BTLA expression Ex vivo-expanded Vγ9Vδ2 T cells were stimulated as described in Figure 1. **(A)** Representative images of cytokine-activated Vγ9Vδ2 T cells. **(B, C)** Representative histogram and geometric mean fluorescence intensity (gMFI) of ICAM-1 **(B)** and CD25 **(C)** by CD3^+^Vδ2 TCR^+^ cells. **(D)** Representative contour plots and percentage of BTLA-positive cells gated on CD3^+^-cells. The data for healthy donors (n = 4) was obtained from independent experiments. Bars represent the mean and SEM, *P < 0.05, **P < 0.01, one-way ANOVA, followed by Tukey's multiple comparisons test.

Antigen-dependent stimulation of T cells is associated with the induction of integrin-mediated adhesion and enhanced expression of adhesion molecules. We examined the ability of IL-12 and IL-18 to enhance the effector function of ex vivo-expanded Vγ9Vδ2 T cells and observed that treatment with IL-12 and IL-18 resulted in the induction of robust homotypic aggregates (Figure 3A). The formation of these aggregates upon treatment with IL-12 and IL-18 was associated with increased levels of ICAM-1 compared with untreated-, IL-12- or IL-18-treated cells (Figure 3B).

Next, to determine whether cytokine treatment influenced other markers of Vγ9Vδ2 T-cell activation, we measured the levels of CD25. Cells treated with IL-12 and IL-18 expressed increased levels of CD25 compared with cells treated with medium, IL-12, or IL-18 (Figure 3C).

The co-inhibitory receptor BTLA is expressed by resting Vγ9Vδ2 T cells, and TCR-mediated activation downregulates its expression on the cell surface [9]. To determine whether ex vivo-expanded Vγ9Vδ2 T cells express BTLA and whether cytokine-mediated activation affects its cell-surface expression, we analyzed BTLA expression by Vγ9Vδ2 T cells treated with medium, IL-12, IL-18, or a combination of both cytokines. More than 40% of untreated- or IL-12-treated Vγ9Vδ2 T cells expressed BTLA on their surface (Figure 3D); however, treatment with IL-18 alone and in combination with IL-12 decreased the numbers of BTLA-expressing Vγ9Vδ2 T cells (Figure 3D). Thus, consistent with antigen stimulation, treating Vγ9Vδ2 T cells with IL-12 together with IL-18 inhibited the expression of BTLA.

### IL-12 and IL-18 augment Vγ9Vδ2 T cell-mediated cytotoxicity against tumor cells

**Figure 4:**
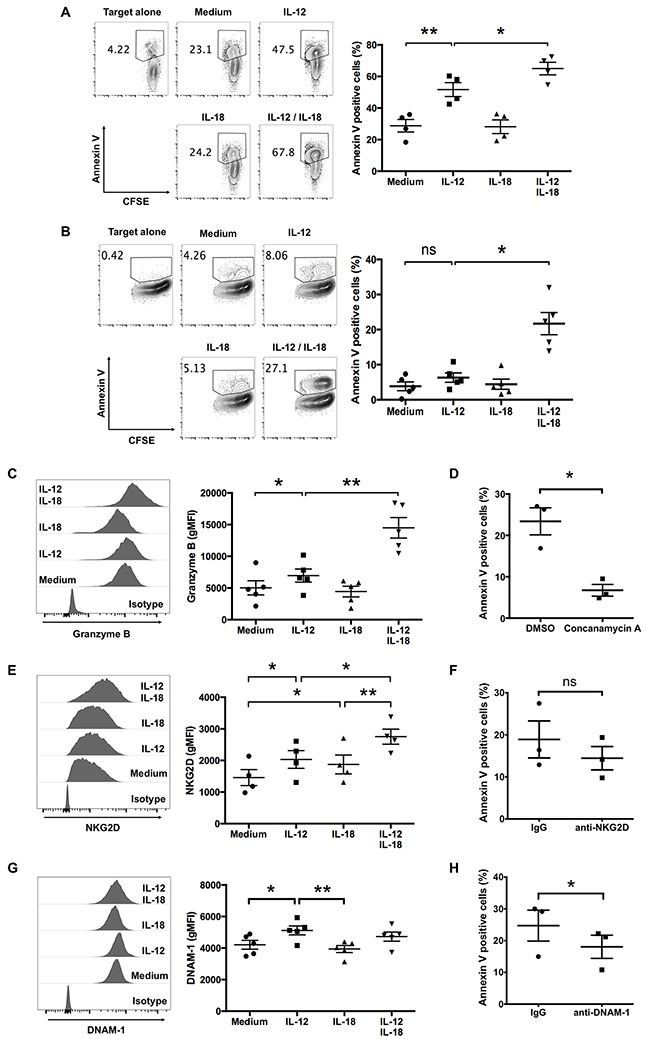
IL-12 alone and in combination with IL-18 augment human Vγ9Vδ2 T cell-mediated cytotoxicity Ex vivo-expanded Vγ9Vδ2 T cells were stimulated as described in Figure 1. CFSE-labeled tumor cells were incubated with activated Vγ9Vδ2 T cells **(A, B)**. **(A)** Representative contour plots and percentage of Annexin V-positive RPMI8226 myeloma cells after 2 h of incubation with cytokine-activated Vγ9Vδ2 T cells (E:T = 4:1). **(B)** Representative contour plots and percentage of Annexin V-positive MG-63 osteosarcoma cells after 4 h of incubation with cytokine-activated Vγ9Vδ2 T cells (E:T = 8:1). **(C, E, G)** Representative histogram and gMFI of granzyme B **(C)**, NKG2D **(E)** and DNAM-1 **(G)** by CD3^+^Vδ2 TCR^+^ cells. The data were obtained from four **(A, E)** or five **(B, C, G)** independent experiments each using Vγ9Vδ2 T cells from different donors. Bars represent the mean and SEM, *P < 0.05, **P < 0.01, one-way ANOVA, followed by Tukey's multiple comparison test. **(D, F, H)** Ex vivo-expanded Vγ9Vδ2 T cells were stimulated with IL-12 and IL-18, then incubated with CFSE-labeled MG63 cells with or without indicated inhibitor **(D)** or blocking antibodies **(F, H)**. The data were obtained from three independent experiments each using Vγ9Vδ2 T cells from different donors. Bars represent the mean and SEM, *P < 0.05, two-tailed paired Student *t* test.

Vγ9Vδ2 T cells exhibit cytotoxicity against multiple types of tumors [19]. Because Vγ9Vδ2 T cells naturally recognize and kill tumors of B-cell origin [16], we examined the effect of cytokine treatment on the cytotoxic activity of ex vivo-expanded Vγ9Vδ2 T cells against RPMI8226 myeloma cells. Although untreated-Vγ9Vδ2 T cells killed >20% of RPMI8226 cells, treatment with IL-12 alone or in combination with IL-18 strongly enhanced cytotoxic activity (Figure 4A).

For another tumor cell target, we used MG-63 osteosarcoma cells, which are poor activators of Vγ9Vδ2 T cells in the absence of an anti-CD277 agonistic antibody (20.1) [3]. As expected, untreated ex vivo-expanded Vγ9Vδ2 T cells did not kill MG-63 cells efficiently; however, IL-12/IL-18 treatment enhanced their cytotoxicity against MG-63 cells (Figure 4B). These results suggest that combined treatment with IL-12 and IL-18 can enhance the cytotoxicity of ex vivo-expanded Vγ9Vδ2 T cells against tumor cells.

Next, we examined the expression level of granzyme B in response to these cytokines. Consistent with the enhanced cytotoxic activity, granzyme B expression in ex vivo-expanded Vγ9Vδ2 T cells increased in response to IL-12 and IL-18 (Figure 4C). We next treated IL-12/IL-18 activated Vγ9Vδ2 T cells with concanamycin A, which attenuates perforin mediated cytotoxicity, before the co-culture with MG-63 cells. As shown in Figure 4D, concanamycin A suppressed the cyototoxic activity of Vγ9Vδ2 T cells against MG-63. Thus, these results suggest that IL-12/IL-18 activated Vγ9Vδ2 T cells kill target tumor cells via secretory granule pathway.

It is reported that the NK receptor NKG2D and DNAX Accessory Molecule-1 (DNAM-1) are expressed on Vγ9Vδ2 T cells and contribute to tumor cell recognition [20]. We examined the effect of IL-12 and IL-18 on the cell surface expression level of these receptors and found that NKG2D, but not DNAM-1, increased slightly in response to combined treatment of IL-12 and IL-18 (Figure 4E, 4G). Since MG-63 cells express the ligands to these receptors (Supplementary Figure 2), we utilized blocking antibodies to evaluate the potential role of these receptors in the recognition of MG-63 target cells. Although anti-NKG2D blocking antibody did not affect the cytotoxic activity of Vγ9Vδ2 T cells against MG-63 target cells, blockade of DNAM-1 slightly reduced the cytotoxicity (Figure 4F, 4H). These results suggest that IL-12/IL-18 activated Vγ9Vδ2 T cells recognize tumor cells, at least in part, via DNAM-1.

### IL-12 and IL-18 synergistically induce IκBζ expression in ex vivo-expanded Vγ9Vδ2 T cells

**Figure 5:**
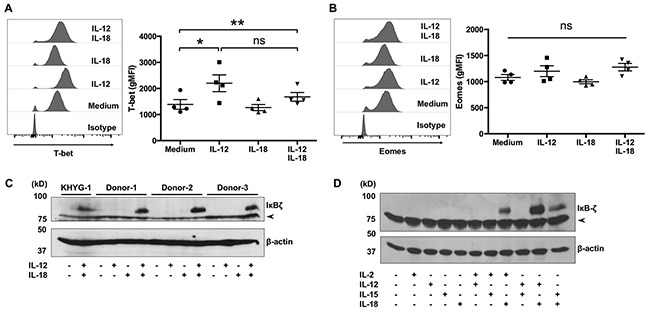
IκBζ is expressed in human Vγ9Vδ2 T cells in response to IL-12 and IL-18 Ex vivo-expanded Vγ9Vδ2 T cells were stimulated as described in Figure 1. **(A, B)** Cells were harvested and analyzed to detect the cell-surface of CD3 and Vδ2 TCR and intracellular expression of transcription factors. Representative histogram and gMFI of T-bet **(A)** and Eomes **(B)** by CD3^+^Vδ2 TCR^+^ cells. Data from healthy donors (n = 4) obtained from independent experiments. Bars represent the mean and standard error of the mean (SEM), **P < 0.01, *P < 0.05, one-way ANOVA, followed by Tukey's multiple comparison test. **(C, D)** Western blot analysis of IκBζ expression in cell lysates prepared from Vγ9Vδ2 T cells and KHYG-1 NK cells which were treated with indicated combinations of cytokines for 16 h. The arrowhead indicates nonspecific band.

The transcription factors T-bet and Eomes are critical for IFN-γ expression by mouse γδ T cells [21, 22]. To determine whether the cytokine-induced production of IFN-γ is associated with these transcription factors, we determined the expression levels of T-bet and Eomes in cytokine-treated Vγ9Vδ2 T cells. Most of the ex vivo-expanded Vγ9Vδ2 T cells expressed T-bet and Eomes (Figure 5A, 5B), and the expression levels of T-bet were slightly increased in response to IL-12 treatment (Figure 5A). Recently, two independent groups reported that IκBζ is indispensable for the induction of IFN-γ by NK cells in response to IL-12 and IL-18 [23, 24]. Therefore, we next determined the expression levels of IκBζ in ex vivo-expanded Vγ9Vδ2 T cells. Although resting, IL-12-treated, or IL-18-treated Vγ9Vδ2 T cells did not express IκBζ, combined treatment with IL-12/IL-18 induced IκBζ expression in Vγ9Vδ2 T cells (Figure 5C). To determine whether the ability of cytokines to induce robust expression of IκBζ in Vγ9Vδ2 T cells is unique to the combination of IL-12 and IL-18, we treated ex vivo-expanded Vγ9Vδ2 T cells with combinations of cytokines including IL-2, IL-12, IL-15 and IL-18. Combined treatment of IL-18 with IL-2 or IL-15 induced IκBζ expression; however, it was not as strong as the combined treatment of IL-12 and IL-18 (Figure 5D).

### IκBζ expression is critical for the production of IFN-γ by ex vivo-expanded Vγ9Vδ2 T cells in response to IL-12 and IL-18

**Figure 6:**
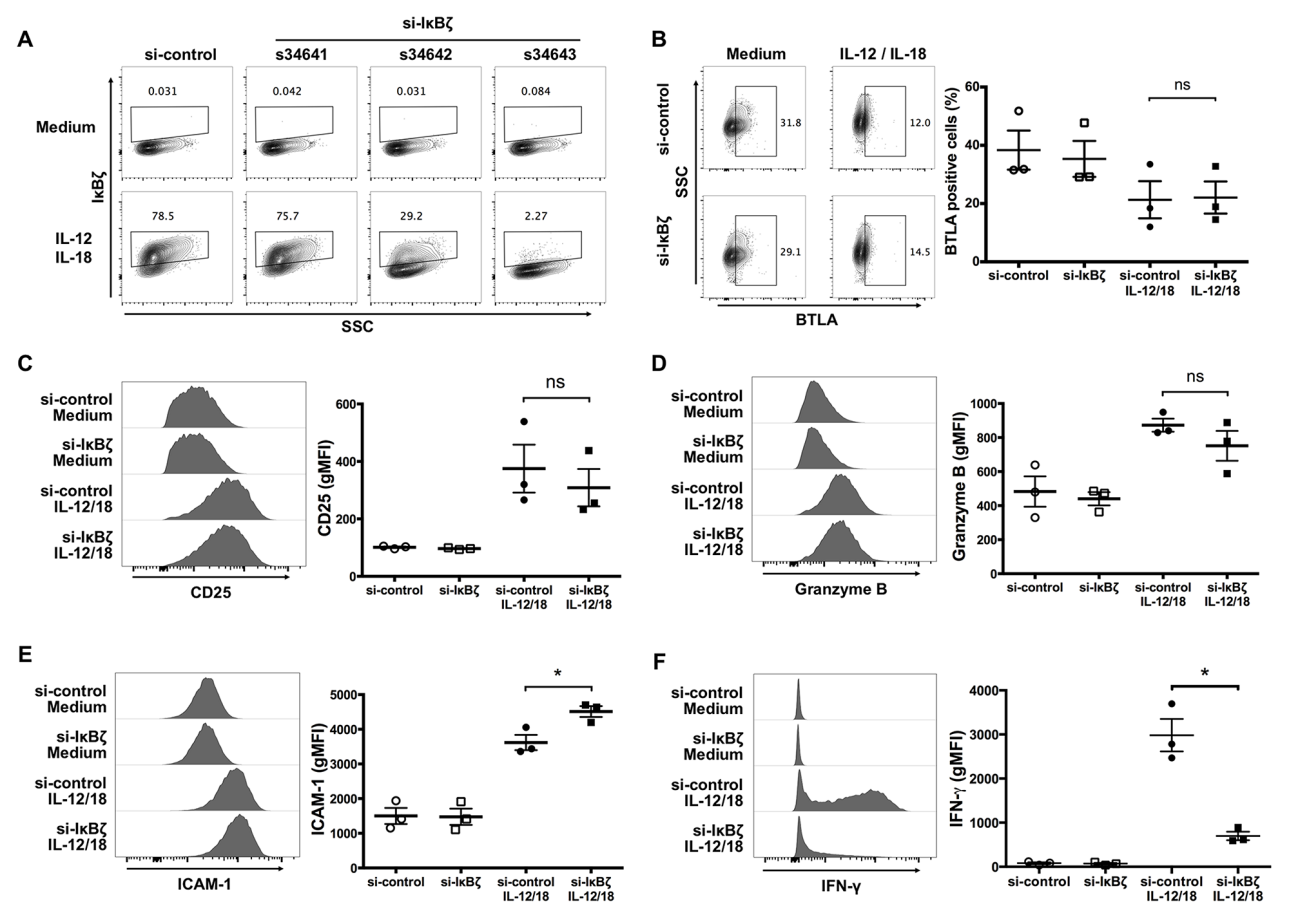
Silencing of IκBζ expression in human Vγ9Vδ2 T cells attenuates IFN-γ production in response to IL-12 and IL-18 Ex vivo-expanded Vγ9Vδ2 T cells were transfected with siRNA targeting IκBζ or control siRNA followed by 16-hour treatment of IL-12 and IL-18. Zombie violet positive cells were gated-out as dead cells. **(A)** Representative contour plots of IκBζ-positive cells gated on CD3^+^ Vδ2 TCR^+^ cells. Among three different siRNAs targeting IκBζ, one (s34643) silenced cytokine-induced IκBζ efficiently, and which was used for knocking down IκBζ in further experiments. **(B)** Representative contour plots and percentage of BTLA-positive cells by CD3^+^Vδ2 TCR^+^ cells. **(C-F)** Representative histogram (left) and gMFI (right) of CD25 **(C)**, granzyme B **(D)**, ICAM-1 **(E)** and IFN-γ **(F)** by CD3^+^Vδ2 TCR^+^ cells. The data were obtained from three independent experiments each using Vγ9Vδ2 T cells from different donors. Bars represent the mean and SEM, *P < 0.05, two-tailed paired Student *t* test.

To determine the significance of IκBζ for the IL-12/IL-18-induced activation of Vγ9Vδ2 T cells, the cells were transfected with siRNA targeting IκBζ by electroporation before the treatment of IL-12 and IL-18. We observed that one IκBζ-targeting siRNA, s34643, efficiently suppressed the induction of IκBζ induced by IL-12/IL-18 treatment (Figure 6A). We co-stained cell surface and intracellular activation markers with IκBζ. We found that silencing of IκBζ did not affect the expression levels of BTLA, CD25 and granzyme B (Figure 6B, 6C, 6D), but did result in a slight increase in ICAM-1 expression (Figure 6E). However, silencing of IκBζ suppressed the robust expression of IFN-γ in response to IL-12 and IL-18 by Vγ9Vδ2 T cells (Figure 6F). Thus, similar to NK cells, IκBζ induced by IL-12/IL-18 is a critical mediator of IFN-γ production by ex vivo-expanded Vγ9Vδ2 T cells.

### IL-12 and IL-18 reciprocally increase their cell surface receptor expression in ex vivo-expanded Vγ9Vδ2 T cells

**Figure 7:**
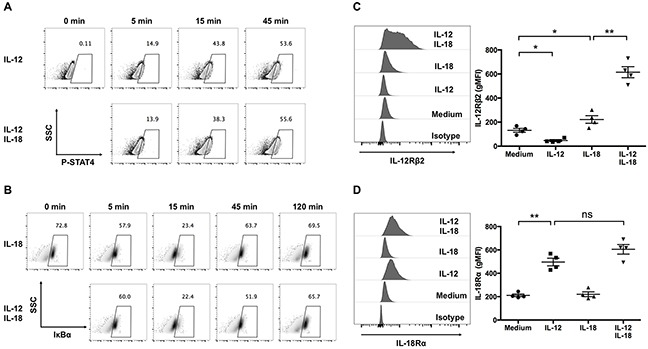
IL-12 increases the expression of IL-18Rα, and in combination with IL-18, synergistically increase the expression of IL-12Rβ2 in human Vγ9Vδ2 T cells **(A, B)** Ex vivo-expanded Vγ9Vδ2 T cells were stimulated with indicated cytokines for different periods of time. Representative contour plots of p-STAT4-positive cells **(A)** and representative density plots of IκBα-positive cells **(B)**. **(C, D)** Ex vivo-expanded Vγ9Vδ2 T cells were stimulated as described in Figure 1. Representative histogram and gMFI of IL-12Rβ2 **(C)** and IL-18Rα **(D)** by CD3^+^Vδ2 TCR^+^ cells. The data for healthy donors (n = 4) was obtained from independent experiments. Bars represent the mean and SEM, *P < 0.05, **P < 0.01, one-way ANOVA, followed by Tukey's multiple comparisons test.

As IL-12 and IL-18 activate the JAK/STAT pathway and the MyD88/TRAF6 pathway, respectively, we investigated whether combined treatment of IL-12 and IL-18 alters these signal transduction pathways in ex vivo-expanded Vγ9Vδ2 T cells. First, we examined the activation status of STAT4, which is phosphorylated by JAKs in response to IL-12. Treatment with IL-12 increased phospho-STAT4 (p-STAT4) positive cells (Figure 7A); however, combined treatment with IL-18 did not alter the p-STAT4 positive population up to 45-min stimulation (Figure 7A). Next, we examined the proteolytic degradation of IκBα, a downstream signaling event of the MyD88/TRAF6 pathway, in ex vivo-expanded Vγ9Vδ2 T cells in response to IL-12/IL-18 treatment. We observed comparable degradation kinetics of IκBα in IL-18-treated or IL-12/IL-18-treated cells (Figure 7B). Thus, the combined treatment of IL-12 and IL-18 did not alter the early proximal signal transduction pathways of each other. It is reported that treatment of T helper cells with IL-12 or IL-18 enhanced the mRNA transcription of IL-18Rα or IL-12Rβ2, respectively [25]. Therefore, we examined the expression levels of IL-12Rβ2 and IL-18Rα on ex vivo-expanded Vγ9Vδ2 T cells after a 16-h stimulation with IL-12 and/or IL-18. Consistent with the previous report, IL-12 and IL-18 increased the cell surface expression of IL-18Rα and IL-12Rβ2, respectively (Figure 7C, 7D). Interestingly, we observed that treatment of IL-18 in combination with IL-12 synergistically increased IL-12Rβ2 expression in Vγ9Vδ2 T cells (Figure 7C).

### IL-12 and IL-18 synergistically induce sustained activation of STAT4 and NF-κB in human ex vivo-expanded Vγ9Vδ2 T cells

**Figure 8:**
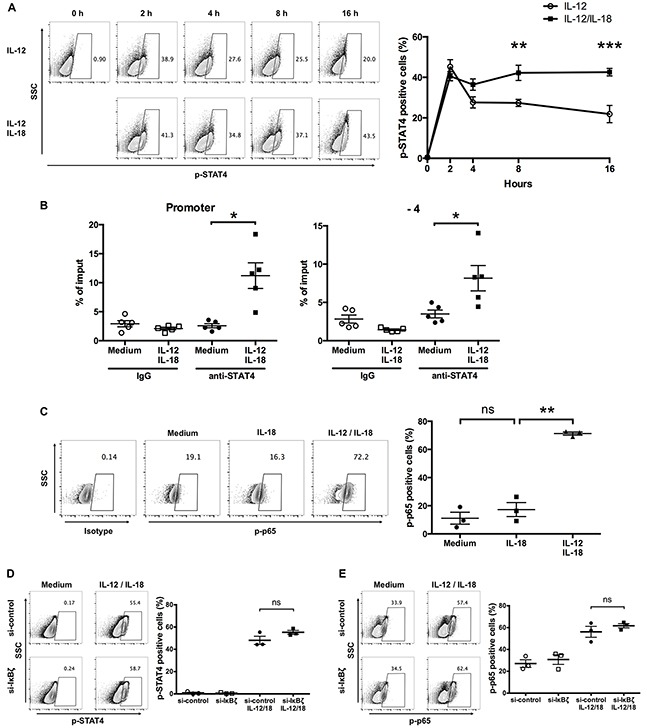
IL-12 and IL-18 synergistically induce sustained phosphorylation of STAT4 and NF-κB p65 in human Vγ9Vδ2 T cells **(A)** Ex vivo-expanded Vγ9Vδ2 T cells were stimulated with IL-12 alone or in combination with IL-18 for different periods. Representative contour plots and percentage of p-STAT4 positive cells. The data for healthy donors (n = 4) was obtained from independent experiments. Bars represent the mean and SEM, **P < 0.01, ***P < 0.001, two-way ANOVA, followed by Sidak's multiple comparisons test. **(B)** Ex vivo-expanded Vγ9Vδ2 T cells were activated with IL-12 and IL-18 for 16 h followed by chromatin immunoprecipitation using anti-STAT4 Ab. Recruitment of STAT4 to the promoter (left) and −4 kb (right) of IFN-γ gene regions were analyzed by qPCR. The data were obtained from five independent experiments each using Vγ9Vδ2 T cells from different donors. Bars represent the mean and SEM, *P < 0.05, two-tailed paired Student *t* test. **(C)** Ex vivo-expanded Vγ9Vδ2 T cells were stimulated with IL-18 alone or in combination with IL-12 for 16 h. Representative contour plots and percentage of p-p65 positive cells. The data for healthy donors (n = 3) was obtained from independent experiments. Bars represent the mean and SEM, **P < 0.01, one-way ANOVA, followed by Tukey's multiple comparison test. **(D, E)** Ex vivo-expanded Vγ9Vδ2 T cells were transfected with siRNA targeting IκBζ or control siRNA followed by 16-hour treatment of IL-12 and IL-18. Representative contour plots and percentage of p-STAT4- **(D)** and p-p65- **(E)** positive cells by CD3^+^Vδ2 TCR^+^ cells. The data were obtained from three independent experiments each using Vγ9Vδ2 T cells from different donors. Bars represent the mean and SEM, two-tailed paired Student *t* test.

Since combined treatment of IL-12 and IL-18 enhanced their cell surface receptor expression, we next examined the activation status of the transcription factors that are activated by IL-12 and IL-18 stimulation. First, to determine if this increased expression of IL-12Rβ2 in response to IL-12/IL-18 exposure affects STAT4 phosphorylation at a later time point, we treated ex vivo-expanded Vγ9Vδ2 T cells with IL-12 alone or in combination with IL-18 up to 16 h. We observed that Vγ9Vδ2 T cells showed about 40% of p-STAT4 positive population after 2-h treatment with IL-12 alone, and the percentage of p-STAT4-positive population declined thereafter (Figure 8A). Although simultaneous treatment of IL-12 and IL-18 resulted in comparable p-STAT4-positive Vγ9Vδ2 T cell population at 2 h post stimulation, the p-STAT4-positive population did not decline thereafter and was sustained at least up to 16 h post stimulation (Figure 8A). We also examined the recruitment of STAT4 to the *IFNG* proximal promoter region by chromatin immunoprecipitation (ChIP)-qPCR analysis. We found that STAT4 was recruited to the promoter and -4kb region of *IFNG* in Vγ9Vδ2 T cells in response to 16 h stimulation with IL-12 and IL-18 (Figure 8B). Next, we examined whether the increased expression of IL-18Rα in response to 16 h IL-12/IL-18 exposure affects the phosphorylation status of NF-κB p65 at this time point. Untreated and IL-18 treated ex vivo-expanded Vγ9Vδ2 T cells showed comparable phosphorylation status of NF-κB p65. In contrast, IL-12/IL-18 treated cells showed increased phosphorylation status of NF-κB p65 (Figure 8C). These results suggest that increased expression of receptors for IL-12 and IL-18 results in the sustained activation of STAT4 and NF-κB. Finally, we examined whether silencing of IκBζ affects the enhanced phosphorylation of STAT4 and NF-κB p65 in Vγ9Vδ2 T cells in response to IL-12/IL-18. IκBζ-silenced Vγ9Vδ2 T cells showed comparable p-STAT4 and p-p65 positive populations compared with that of control cells (Figure 8D, 8E). Thus, the prolonged activation of STAT4 and NF-κB p65 in Vγ9Vδ2 T cells in response to IL-12/IL-18 is independent of IκBζ expression.

## DISCUSSION

In addition to NK or T cell receptor activation, NK cells and αβ T cells can respond to certain combinations of cytokines, particularly IL-12 and IL-18 [26, 27]. As Vγ9Vδ2 T cells respond to IL-12 or IL-18 in combination with antigen stimulation [8], we hypothesized that Vγ9Vδ2 T cells respond to IL-12 and IL-18 independent of NK or T cell receptor activation. Here, we demonstrated that the combination of IL-12 and IL-18 induces human Vγ9Vδ2 T cell activation independent of the presence of antigen or activation of the NK receptor.

IL-12 and IL-18 synergize to induce IFN-γ expression by multiple lymphocyte subsets, including human T cells [28], human CD4^+^ T cells [29], human CD8^+^ T cells [30], human NK cells [31], murine NK cells [14], murine CD4^+^ T cells [32], and murine CD8^+^ T cells [33]. IFN-γ is a Th1 cytokine that is critical for innate and adaptive immune responses, and is produced by Vγ9Vδ2 T cells in response to antigen stimulation [16]. Moreover, our results demonstrated that exposure to IL-12 and IL-18 induces robust IFN-γ expression by ex-vivo expanded Vγ9Vδ2 T cells. We also demonstrated that IL-12 and IL-18 induced IFN-γ expression in naïve Vγ9Vδ2 T cells. These results suggest that activation of Vγ9Vδ2 T cells by the combination of IL-12 and IL-18 does not require antigen stimulation. This is consistent with the characteristics of Vγ9Vδ2 T cells which are considered as innate-like T lymphocyte with NK cell characteristics [34].

Previous studies of NK and αβ T cells suggest that in addition to enhanced production of effector molecules and increased cytotoxicity, these cells are induced to proliferate upon exposure to a combination of IL-12 and IL-18 [13, 27]. Our present results demonstrated that ex vivo-expanded Vγ9Vδ2 T cells, but not freshly isolated Vγ9Vδ2 T cells, proliferate in response to combined exposure to IL-12 and IL-18 and that this response is enhanced by subsequent treatment with low concentrations of IL-2 or IL-15.

Vγ9Vδ2 T cells kill a broad range of tumors, including breast cancers [35, 36], colon carcinomas [37], lymphomas [38], melanomas [39], myelomas [16], and prostate cancers [40]. These tumors are particularly recognized by Vγ9Vδ2 T cells via NK receptors and the Vγ9Vδ2 TCR. Using myeloma and osteosarcoma cell lines as targets, we demonstrated here that the treatment of ex vivo-expanded Vγ9Vδ2 T cells with IL-12 and IL-18 enhanced their cytotoxic activity against tumor cell lines. We also demonstrated that Vγ9Vδ2 T cells recognize target cells by DNAM-1. Blocking antibodies specific for DNAM-1 significantly blocked Vγ9Vδ2 T cell mediated cytotoxic activity against MG-63 cells. Since the antibody treatment did not completely block the cytotoxic activity, other receptors may contribute to the recognition of tumor cells by IL-12/IL-18 activated Vγ9Vδ2 T cells. Thus, using cytokines to regulate the functions of Vγ9Vδ2 T cells might represent an important aspect of applying ex vivo-expanded Vγ9Vδ2 T cells to cancer therapy.

Recent insights into the mechanism underlying IL-12- and IL-18-induced IFN-γ production by NK cells reveal that the induction of IκBζ is indispensable for IL-12- and IL-18-mediated IFN-γ production by human [23] and murine [24] NK cells. Miyake et al. found that IL-12/IL-18 treatment increases IκBζ mRNA expression by mouse NK cells but not by mouse naïve T cells, Th1 cells, or Th2 cells [24]. Kannan et al. found that treating human lymphocyte with IL-12/IL-18 increased IκBζ expression by CD56^+^CD14^−^ cells but not by CD56^−^/CD14^−^ cells [23]. These findings suggest that the induction of IκBζ in response to the combination of IL-12 and IL-18 is unique to NK cells. However, in this study, we demonstrated that ex vivo-expanded Vγ9Vδ2 T cells induce IκBζ expression in response to IL-12/IL-18 as well, and silencing of IκBζ attenuates the robust induction of IFN-γ. In contrast to the critical role for the regulation of IFN-γ, silencing of IκBζ did not affect the expression levels of granzyme B, CD25, and BTLA in IL-12/IL-18-treated Vγ9Vδ2 T cells. Therefore, we explored other mechanisms underlining the cytokine-mediated activation of ex vivo-expanded Vγ9Vδ2 T cells. We showed that Vγ9Vδ2 T cells treated with IL-12 or IL-18 enhanced the surface expression of IL-18Rα and IL-12Rβ2, respectively, and this is consistent with the previous report that IL-12 or IL-18 reciprocally induced their receptors in Th cells [25]. Moreover, our results showed that IL-12 and IL-18 synergistically increased IL-12Rβ2 expression in Vγ9Vδ2 T cells compared with that by IL-18 treatment alone. We propose that this increased cell surface expression of IL-12Rβ2 and IL-18Rα may result in more efficient cytokine signaling. To test this hypothesis, we examined the phosphorylation status of STAT4 and NF-κB p65 in ex vivo-expanded Vγ9Vδ2 T cells treated with IL-12 and/or IL-18. Our results demonstrated that although both IL-12 alone or IL-12/IL-18 treatment resulted in comparable activation of STAT4 in Vγ9Vδ2 T cells, the duration of activation of STAT4 was longer in IL-12/IL-18-treated cells than in cells treated with IL-12 alone. Further, p-p65 NF-κB positive populations in IL-12/IL-18-treated cells are higher than that of IL-18 alone-treated cells. These results might reflect the enhanced surface expression of IL-12Rβ2 and IL-18Rα on IL-12/IL-18 treated cells. These results suggest that IL-12 and IL-18 treatment synergistically activate ex vivo-expanded Vγ9Vδ2 T cells through the induction of IκBζ, and increased expression of IL-12Rβ2 and IL-18Rα, resulting in sustained activation of STAT4 and NF-κB.

In conclusion, we demonstrate that the combined treatment of IL-12 and IL-18 regulates multiple effector functions of Vγ9Vδ2 T cells, and the induction of IκBζ and prolonged activation of STAT4 and NF-κB are key regulators of the cytokine activation of ex vivo-expanded Vγ9Vδ2 T cells. Thus, our data reveal a novel technique and molecular basis for regulating the phenotype of ex vivo-expanded Vγ9Vδ2 T cells that can be clinically exploited for enhancing the efficacy of tumor immunotherapy.

## MATERIALS AND METHODS

### Ethics

Peripheral blood from healthy volunteers was obtained after obtaining written informed consent from them with the approval of the Research Ethics Committee of Osaka Dental University.

### Preparation of ex vivo-expanded Vγ9Vδ2 T cells

Peripheral blood mononuclear cells (PBMCs) were isolated from fresh blood by density gradient centrifugation using a BD Vacutainer CPT (BD Biosciences, San Jose, CA). PBMCs were cultured at 37°C and 5% CO_2_ in 24-well plate at a density of 1 × 10^6^ cells/mL in RPMI 1640 (Wako, Japan) that was supplemented with 10% FBS (Sigma–Aldrich, St. Louis, MO), 4 mM L-glutamine, 100 U/mL penicillin, 100 μg/mL streptomycin (Nacalai tesque, Japan), 100 U/mL IL-2 (Primmune, Japan), and 5 μM zoledronic acid (Cayman Chemical, Ann Arbor, MI). To expand Vγ9Vδ2 T cells, additional IL-2 (100 U/mL) was added on day 3 to prevent IL-2 starvation, and the culture density was maintained at 0.5 to 2 × 10^6^ cells/mL by adding fresh medium supplemented with IL-2 (100 U/mL). On day 14, the expanded cells were collected and extensively washed with medium; thereafter, they were cryopreserved for further experiments. The purity of Vγ9Vδ2 T cells was >93%.

### Isolation of Vγ9Vδ2 T cells from fleshly prepared PBMC

Untouched Vγ9Vδ2 T cells were enriched from fleshly isolated PBMC using γ/δ^+^ T cell isolation kit, human (Miltenyi Biotec, Auburn, CA) according to the manufacture's protocol. The purity of Vγ9Vδ2 T cells was >85%.

### Cell culture

The KHYG-1 NK cell line was provided by H. Umehara [41]. RPMI8226 myeloma and MG-63 osteosarcoma cell lines were obtained from the RIKEN BioResource Center (Tsukuba, Japan) and cultured in RPMI-1640 or DMEM, each of which were supplemented with 10% FBS, 4 mM L-glutamine, 100 U/mL penicillin, and 100 μg/mL streptomycin. For cytokine-mediated activation of Vγ9Vδ2 T cells, cryopreserved cells were thawed and extensively washed with medium; thereafter, they were cultured for indicated period with IL-2 (100 U/mL), IL-12 (10 ng/mL) (BioLegend, San Diego, CA), IL-15 (10 ng/mL) (BioLegend), IL-18 (10 ng/mL) (MBL International, Woburn, MA), or two out of them.

### Flow cytometry (FACS)

Cells were labeled with the monoclonal antibodies as follows: CD3 (clone OKT3) and ICAM-1 (clone 15.2) from Tonbo Biosciences (San Diego, CA); TCR Vδ2 (clone B6), TCR Vγ9 (clone B3), CD25 (clone BC96), CD27 (clone O323), CD45RA (HI100), BTLA (clone MIH26), NKG2D (clone 1D11), DNAM-1 (clone 11A8), and IL-18Rα (clone H44) from BioLegend; IL-12Rβ2 (REA333) from Miltenyi Biotec. For detecting transcription factor expression, cells were surface-stained, fixed, permeabilized using the Foxp3/Transcription Factor Staining Buffer Set (eBioScience, San Diego, CA), and stained with anti-T-bet (clone REA102) (Miltenyi Biotec), anti-Eomes (clone WD1928) (eBioscience) and anti-IκBζ (clone hft2nap) (eBioscience) antibodies. For intracellular staining, monensin (BioLegend) was added during the last 2 h of culture. After staining with antibodies to detect cell surface antigens, cells were fixed and permeabilized using Fixation and Perm/Wash buffer (BioLegend) and then treated with anti-IFN-γ (clone 4S. B3) and granzyme B (clone GB11) antibodies (both from BioLegend). For examining kinetics of signal transduction molecules, cytokine treated cells were fixed immediately by adding IC Fixation buffer (eBioscience) followed by particular permeabilization methods. For detecting intracellular IκBα, fixed cells were permabilized with Perm/Wash buffer (BioLegend) followed by incubation with anti-IκBα antibody (clone MFRDTRK) (eBioscience). For detecting intracellular p-STAT4 or p-p65, fixed cells were permabilized with ice cold methanol followed by incubation with anti-p-STAT4 (Y693) antibody (clone 4LURPIE) (eBioscience) or anti-p-NF-κB p65 (S536) antibody (clone 93H1) (Cell Signaling Technology, Danvers, MA). Data were collected using a BD FACSVerse (BD); the resulting data were analyzed using FlowJo software X 10.0.7 (Tree Star, Ashland, OR).

### Cell proliferation assay

Vγ9Vδ2 T cells were labeled with 2 μM CTV (Life Technologies, Grand Island, NY) and treated with IL-12, IL-18, or both for 16 h. After extensive washing, Vγ9Vδ2 T cells were further cultured with medium alone or with medium supplemented with IL-2, IL-7 (BioLegend), or IL-15 for an additional 3 days. CD3^+^Vδ2 TCR^+^ cells were gated, and CTV-diluted cells were counted as dividing cells. In some experiments, labeled Vγ9Vδ2 T cells were stimulated with plate bound anti-CD3 (clone UCHT1) and soluble anti-CD28 (clone CD28.2) (both from BioLegend) and used for positive control.

### In vitro tumor killing assay

Ex vivo-expanded Vγ9Vδ2 T cells were activated as described above and extensively washed with medium to remove cytokines. RPMI8226 myeloma cells were labeled with 1 μM carboxyfluorescein succinimidyl ester (CFSE) (Dojindo), and 1 × 10^5^ CFSE-labeled tumor cells were incubated with 4 × 10^5^ Vγ9Vδ2 T cells in RPMI-1640 for 2 h in a 24-well plate. MG-63 osteosarcoma cells were cultured overnight at a density of 6 × 10^4^ cells per well in a 48-well plate and then labeled with 1 μM CFSE before incubation with 4.8 × 10^5^ Vγ9Vδ2 T cells in RPMI-1640 for 4 h. Cells were collected and stained with Annexin V-APC (BioLegend) and subjected to FACS analysis. CFSE^+^Annexin V^+^ cells were considered to be dead tumor cells. In some experiments, IL-12/IL-18 activated Vγ9Vδ2 T cells were treated with 100 ng/mL of concanamycin A (Santa Cruz Biotechnology, Dallas, TX), 10 μg/mL of anti-NKG2D (clone 1D11) (BioLegend) or 10 μg/mL of anti-DNAM-1 (clone 102511) (R&D Systems, Minneapolis, MN) for 30 min before the coincubation with MG-63 cells.

### Western blotting

Ex vivo-expanded Vγ9Vδ2 T cells were activated as described above and lysed directly in sample buffer. Samples were sonicated and boiled for 3 min and then separated using 8% SDS-PAGE with 6 × 10^5^ cell equivalents loaded per lane. The proteins were transferred to PVDF membranes, which were blocked with TBS containing 5% BSA and 0.1% Tween 20, and probed with rabbit anti-human IκBζ (#9244) (Cell Signaling Technology) or mouse anti-β actin (clone AC-74) (Sigma–Aldrich, St. Louis, MO) antibodies, followed by incubation with HRP-conjugated secondary antibodies (GE Health Care Life sciences, U.K.). The chemiluminescence signal was generated using SuperSignal West Pico Chemiluminescent Substrate (GE Health Care Life sciences) and detected using Amersham Hyperfilm ECL (GE Health Care Life sciences).

### siRNA transfection

Transfection of siRNA was conducted using Amaxa Nucleofector device and human T cell nucleofector kit (Lonza, Basel, Switzerland) according to the manufacture's protocol. Briefly, cryopreserved ex vivo-expanded Vγ9Vδ2 T cells were thawed and incubated in complete medium for 2 h; 5 × 10^6^ Vγ9Vδ2 T cells were transfected with 300 nM of negative control or IκBζ targeting (s34641, s34642, or s34643) siRNAs (silencer select, Thermo Fisher scientific, Rockford, IL) with device's program T-20. Transfected cells were cultured in complete medium for 4 h then used for further experiments.

### Chromatin immunoprecipitation (ChIP)

Ex vivo-expanded Vγ9Vδ2 T cells that were stimulated with IL-12/IL-18 for 16 h were cross-linked with 1% formaldehyde for 10 min at room temperature, followed by incubation with 125 mM glycine. Cells were harvested, washed with PBS and re-suspended in 0.5 mL of ChIP buffer (10 mM HEPES-KOH (pH 7.9), 200 mM KCl, 1mM CaCl_2_, 1.5 mM MgCl_2_, 5% sucrose and 0.5% NP-40) containing 10 μM N-(Methoxysuccinyl)-Ala-Ala-Pro-Val-chloromethyl-ketone (Sigma–Aldrich), 1mM sodium orthovanadate (New England Biolabs, Ipswich, MA) and protease inhibitor cocktail (Nacalai tesque). The lysates were incubated on ice for 15 min and sonicated for 5 s three times, followed by digestion with 2 μL of Micrococcal Nuclease (New England Biolabs) and 2 μL of 10 mg/mL RNase A (Thermo Fisher scientific) for 40 min at 37°C. After adding 5 μL of 0.5 M EDTA, the digested samples were centrifuged at 20,000 × *g* for 10 min. The supernatants were incubated with anti-STAT4 antibody (clone EP1900Y, Abcam, Cambridge, MA) / dynabeads protein G (Thermo Fisher scientific) complexes at 4°C overnight. The immunoprecipitated complexes were washed twice with ChIP buffer and twice with TE buffer. After that, cross-links were reversed by incubating in ChIP direct elution buffer (10 mM Tris-HCl (pH 8.0), 300 mM NaCl, 5 mM EDTA and 0.5% SDS) for 6 h at 65°C. After incubation with 50 μg/mL of proteinase K (Thermo Fisher scientific) for 1 h at 55°C, DNA was purified by phenol-chloroform extraction and ethanol precipitation using Dr. GenTLE Precipitation Carrier (TaKaRa, Japan). Quantitative real-time PCR was performed by Thunderbird SYBR qPCR Mix (Toyobo, Japan) and the products were detected with StepOnePlus Real-Time PCR System (Thermo Fisher scientific). The primers were purchased from Cell Signaling Technology (SimpleChIP Human IFN-γ promoter primers #13051) and QIAGEN (EpiTect ChIP qPCR Assay GPH1017290 (−4kb)). Each ChIP value was normalized by the input.

### Statistical analysis

Statistical analysis was performed using GraphPad Prism software 6.0 for Mac OS X (GraphPad Software, La Jolla, CA). Two-tailed Student *t* test or One-way ANOVA followed by Tukey's or Dunnett's multiple comparison tests were used to assess the statistical significance of the differences between data.

## SUPPLEMENTARY FIGURES


