# Supplementary material for: Cytokine-mediated activation of human ex vivo-expanded Vγ9Vδ2 T cells
Source: Oncotarget. 2017 Apr 28;8(28):45928–42. doi: 10.18632/oncotarget.17498 (PMC5542238; doi:10.18632/oncotarget.17498)
Supplement: Supplementary file 1 [file oncotarget-08-45928-s001.pdf]

Cytokine-mediated activation of human ex vivo-expanded Vγ9Vδ2 T cells

SUPPLEMENTARY FIGURES

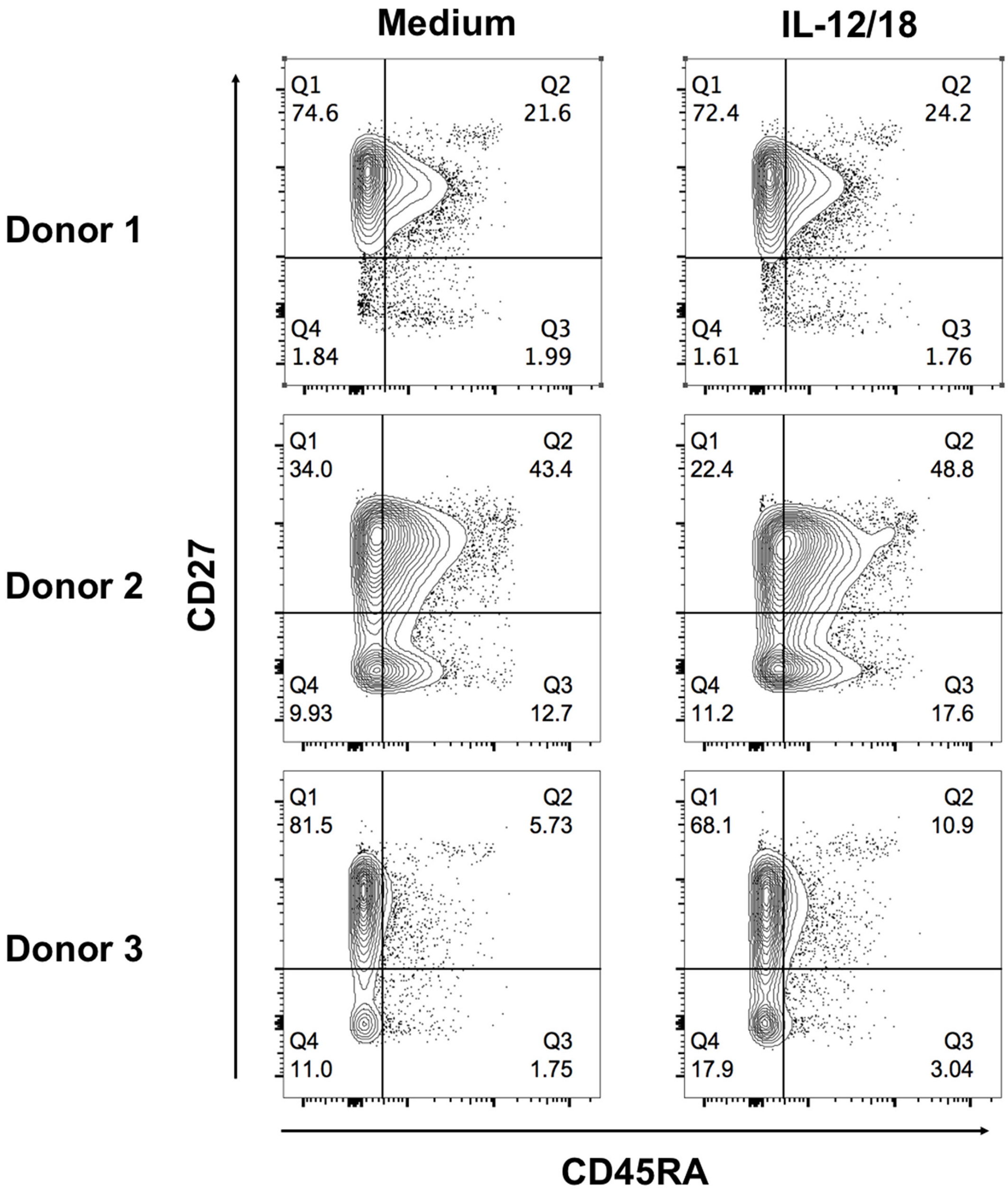

Supplementary Figure 1: Freshly isolated Vγ9Vδ2 T cells consist of antigen experienced and unexperienced cells. Vγ9Vδ2 T cells were isolated from PBMC prepared from three donors. Cells were treated or untreated with IL-12/IL-18 for 16 h. Cell surface expression of CD27 and CD45RA were examined by FACS.

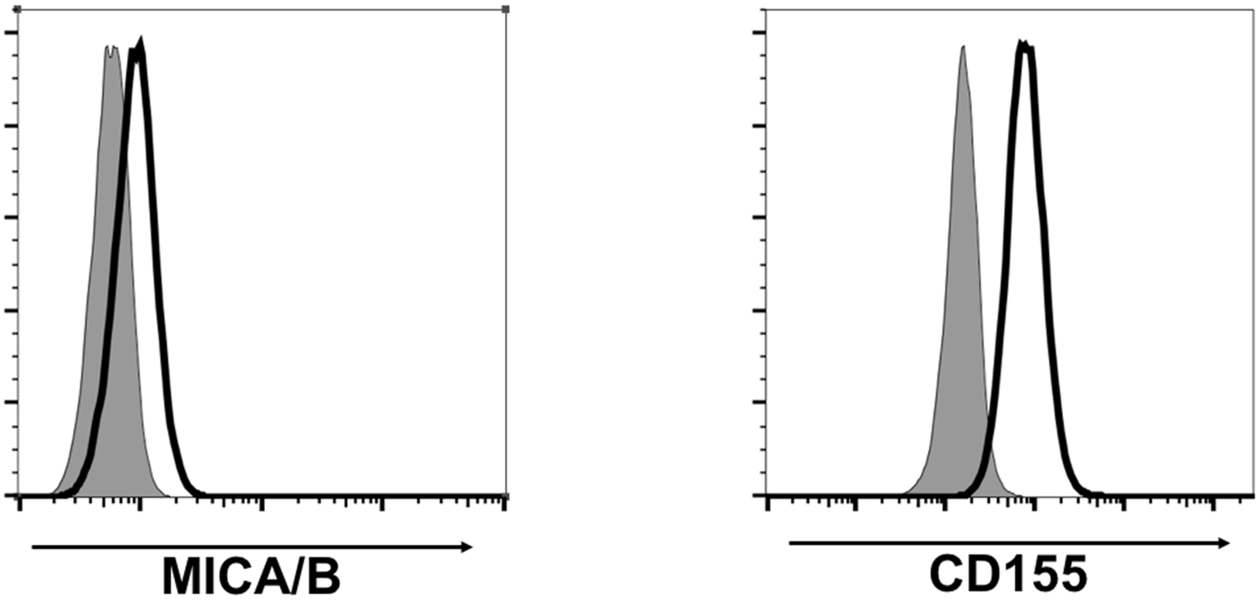

Supplementary Figure 2: MG-63 cell line express MICA/B and CD155. Cell surface expression of MICA/B and CD155 on MG-63 cells were examined by FACS. Tinted: isotype control, heavy line: MICA/B or CD155.
